# Reports on the Hospitals of the United Kingdom

**Published:** 1912-02-10

**Authors:** Henry Burdett


### REPORTS ON THE Hospitals of the United Kingdom.

By SIR HENRY BURDETT, K.C.B., K.C.V.O.

### LXII.—The Royal Hospital, Sheffield.

Judged by its work and the quality of that work, this should be an important hospital. It contains from 170 to 200 beds, and is remarkable that, whilst administratively it can claim to occupy a deservedly high place amongst British hospitals, structurally it proves to demonstration the wastefulness of attempting to design and extend hospital buildings in the absence of sufficient technical and administrative knowledge of hospital construction. To put it briefly, this hospital may have some interest structurally for the expert if and when it is pulled down and rebuilt with knowledge and reasonable economy in the use of bricks, stone, and mortar. For waste of space and consequent avoidable cost of upkeep we believe this hospital to be almost, if not quite, unique amongst institutions of its size. It would be interesting and profitable to learn why this hospital has failed to secure expert guidance before sanctioning some of the many additions which have been made to the buildings in recent years. For the expert, or for those who are anxious to become experts by acquiring an accurate knowledge of what to avoid in hospital construction, a careful study of the whole of these buildings should prove intensely interesting.

### Some Grave Structural Defects.

It is unusual to find a hospital containing buildings erected within recent years where the essential necessity of securing as few resting-places as possible for dust and impurities has not only been disregarded, but where the opposite policy of manufacturing such disease-generating centres is presented in several parts of this hospital. Again, in the latest wing, which is now in course of construction, although the ward unit so far as the attendant offices go promises to be satisfactory, the ward itself puts hospital construction back many decades. Thus the ceilings on each floor are intersected with beams or girders, an arrangement calculated to harbour many undesirable things, the windows are carried up to the top of the ward, thereby removing the old defect of a reservoir there for stagnant air; but for some unexplained reason this stagnant chamber has been transferred to the lower portion of each ward, where some four or five feet of solid brickwork is placed before commencing the windows. The effect of the plan pursued is to turn the worst kind of ward unit of former years upside down. Not content with this anomaly, those responsible for the design have put a window or windows down one side of the ward, the effect of which will be to multiply corners and points of advantage for the home of undesirable things. The possibility of placing the beds between the windows so as to aid the freer circulation of air is thus destroyed. The new ward contains no provision, so far as we could find, for the admission of air at the floor level, so as to obviate as far as possible the manifest inconveniences, to say the least, of the closed-box system which is here reintroduced after having been condemned and discontinued through many decades. In making these remarks we are not blaming anyone directly, because we have no actual knowledge of those responsible for the numerous missed opportunities to make the buildings modern and up to date.

### Serious Overcrowding.

Another feature of the hospital which we consider should be changed without delay is the serious overcrowding of the children's ward. It is placed at the top of the building and contains thirty-two cots, when the ward was planned to contain twenty beds only. The marked difference in the atmosphere of the crowded children's ward and the other wards of similar size with 20 beds, was serious. Further than this, it is not right for the medical staff to permit the centre of the children's ward to be occupied by a number of additional cots or to have the cots at the side so close together as they are at present, especially at the bottom end of the ward. If children are taken into a general hospital they should be given the maximum of hygienic surroundings, and nothing should be permitted, by which the vitality of the children may be lowered, like the present overcrowding of this children's ward at Sheffield. We learnt that the practice began because more than one child was often placed formerly in the same cot, a most unjustifiable proceeding leading in the direction of the ancient plan of four patients in a bed in the old Hôtel Dieu in Paris. This overcrowding is not fair to the children or their friends, and is most unjust to the nursing staff. There should be a spirit of responsibility demonstrably present in every modern hospital in this country so that no patients shall be admitted for whom the best possible accommodation cannot be provided. A well-administered hospital lays itself out to relieve the greatest number of patients with the fewest number of beds in the shortest possible time. If there is no room, the authorities should publicly state the fact and rely on public support to provide adequately for the increasing number of patients requiring admission. In a hospital where the general administration and the spirit of the workers is so high as at the Royal Hospital, Sheffield, it must be grievous for those who have to do the work at present in the children's ward, where the conditions are not what they ought to be, if due regard is to be paid to the best interests of the patients and the staff.

There is one point of historical interest to which attention may be called. The old "Nightingale" window between the ward and the Sister's room is here oval in shape, and placed at such a height from the floor level that it is not possible for a woman of average height to see the whole ward from it. A truly remarkable instance of an idea, originally meant to minimise the dangers and difficulties of too small a staff, degenerating into a mere hole in the wall, which for practical purposes has had from the first slight if any practical value. Nowadays, when the nursing staff has been so largely increased, and the aim of the best-administered hospitals is so to minimise the hours of duty that a sister, like her nurses, may be able to be really at work whilst she is on duty, the first consequence has been to abolish the "Nightingale" window altogether, and the latest is to omit the Sister's room from the ward unit and to utilise the space for the clinical room.

A High Standard of Efficiency.

Apart from the buildings, this hospital is one which deserves to be thoroughly commended for the high standard of personal work maintained in all departments. All the ward work was proved by a detailed and careful inspection to be carried out with the highest efficiency. Such high efficiency is not easy in a hospital situated in the centre of a large town where the atmosphere is relatively clear and has the maximum of freedom from smoke, carbon, and dirt. At Sheffield the atmosphere is never really pure in this sense, owing to the extraordinary amount of coal consumed and the resulting volumes of smoke, which on a clear day in bright sunshine is seen to hang round the houses and buildings, as a visit to the hospital roof demonstrably proved. The constant increase in the volume of work to be done, and the difficulty of maintaining a high standard of cleanliness and smartness must consequently attain nearly, if not quite, the maximum. Yet it is but the truth to say that throughout the hospital its wards, its offices, its passages and, generally, all the work is so thoroughly done that the whole place was smart and beautifully kept on the day of our visit (September 21, 1911). The same remark applies to the high standard of hygienic efficiency which the surgeons unitedly combine to secure. Their work and the value of the hospital cannot, however, at present reach their highest effectiveness, because the hospital does not possess, as at Leeds, for instance, a semi-convalescent home of at least fifty beds, to which surgical patients can be removed into the best air, directly the moment arrives when such removal must tend to quicken recovery. It would also secure that the bread-winner when discharged would be sent home in a condition of health to enable him to return at once to his employment. There are many wealthy and generous men resident or interested in Sheffield. What Leeds citizens have done for the working classes who need hospital care can surely be done speedily for Sheffield.

Defective Accommodation for the Nurses.

One of the saddest things about the hospitals of Sheffield is the evidence they afford that for some unexplained reason no one of high official position in the management, apart from the resident staff, seems to have recognised not only the imperfect but the actually cruel absence of up-to-date and ample provision for the comfort, recreation, and hygenic security of the resident staff, and especially of the nurses and servants. Where there is a nursing home so-called it has never really contained sufficiently ample or hygienically satisfactory arrangements for the nursing staff. Yet that staff in Sheffield's two royal hospitals at any rate, and in the Children's Hospital, have to our knowledge maintained for twenty years, and still maintain, the highest standard of efficiency in their personal work. The accommodation for the nurses at the Children's Hospital is the best, and is reasonably good, but at the Royal Hospital and the Royal Infirmary, Sheffield, this important feature in hospital efficiency, maintenance and atmosphere is absent. It is our duty to state plainly that the very effect of such a condition of affairs as at present prevails on this point at the two principal hospitals of Sheffield demands, and should receive, immediate attention. To be specifically accurate in the case of the Royal Hospital we have to point out that the so-called nursing home, if any regard is to be had to the number of the nursing staff and their requirements, is wrongly named, for it only at present accommodates the night nurses or some of them. It is not right in the present day, in a big general hospital where the number of surgical cases is ever growing, and the work of the nursing staff is increasingly onerous, that there should be any less provision for members of the nursing staff than a good and comfortably equipped bedroom for each nurse, with ample recreation, commissariat, sitting and dining hall provision, as well as space for those probationers who are undergoing instruction.

Proper House Room for the Resident Staff.

We are bound to believe, having regard to the general efficiency of the administration so far as the patients are concerned, that the managers of this hospital desire to make their institution as admirable as possible for the purposes it was established to fulfil. Nevertheless, for some unexplained reason, possibly the absence of one devoted man as chairman or president in authority, who is so interested in the work and reputation of his hospital that he devotes adequate time to its development and oversight, this hospital exhibits an absence of thoughtful care for the comfort of its resident staff, which we witnessed with amazement. It is but the truth to say, as the result of our careful inspection of the whole establishment, that we discovered no evidence, but the contrary, of any thoughtful care for the comfort not only of the nursing staff but of the whole resident staff from the highest to the lowest. We mention the fact with a wholehearted and earnest purpose to secure that this defect shall be removed promptly. No modern hospital can be regarded as properly administered or entitled even to the maximum of public support until these defects and injustices have been removed. The healthy and vigorous cheerfulness of every man and woman resident and working continuously in the atmosphere of a hospital can alone be maintained by the provision not only of a liberal and varied diet, but by the best, most cheerful, and comfortable housing.

